# Prolonged mask wearing does not alter the oral microbiome, salivary flow rate or gingival health status – A pilot study

**DOI:** 10.3389/fcimb.2022.1039811

**Published:** 2022-11-10

**Authors:** Sheralyn Au, Divyashri Baraniya, Jason Dao, Shehar Bano Awan, Jenelle Alvarez, Shari Sklar, Tsute Chen, Sumant Puri, Nezar N. Al-Hebshi

**Affiliations:** ^1^ Kornberg School of Dentistry, Temple University, Philadelphia, PA, United States; ^2^ Oral Microbiome Research Laboratory, Department of Oral Health Sciences, Kornberg School of Dentistry, Temple University, Philadelphia, PA, United States; ^3^ Department of Restorative Dentistry, Kornberg School of Dentistry, Temple University, Philadelphia, PA, United States; ^4^ Department of Microbiology, Forsyth Institute, Cambridge, MA, United States

**Keywords:** COVID-19, high-throughput nucleotide sequencing, masks, microbiota, mouth, xerostomia

## Abstract

The COVID-19 pandemic has resulted in the widespread use of N95 respirators and surgical masks, with anecdotal reports among healthcare providers and the public of xerostomia, halitosis, and gingivitis, a consortium of symptoms colloquially termed “mask mouth”. However, this has not been scientifically verified. The aim of this study was to assess changes in salivary flow rate, gingival health status and oral microbiome associated with prolonged mask use. A total of 25 dental students (mean age = 26.36 ± 1.58) were included in the study and evaluated at three time points: T1, at the end of at least 2 months of full-day mask wear (7.26 ± 1.56 hours/day); T2, at the end of a period of minimal mask use (1.13 ± 1.13 hours/day); and T3, at the end of 2-3 weeks of resuming full-day mask wear (6.93 ± 1.80 hours/day). Unstimulated whole saliva (UWS) flow rate, xerostomia (on a quantitative scale of 10), gingival index (GI) and plaque index (PI) were assessed at each time point. The salivary microbiome was characterized using 16S rRNA gene sequencing. Overall, UWS flow rates were normal (mean of 0.679 ml/min) and xerostomia, PI and GI scores were low (Mean of 3.11, 0.33 and 0.69, respectively) with no significant differences as a result of prolonged mask wearing. Similarly, there were no significant microbial changes at a false discovery rate (FDR) ≤ 0.05. However, some trends were identified using a nominal p-value cut-off of ≤ 0.01, namely *Gemella sanguinis*, *Streptococcus* sp. Oral taxon 066 and Oral taxon 058 were associated with prolonged mask wear. Trends were also seen by gender, race and age, for example an increase in *P. gingivalis and P. intermedia* with age. In conclusion, we found no evidence that prolonged mask wear adversely affects oral health. The findings support that the oral microbiome of healthy individuals is resilient.

## Introduction

The human oral cavity is colonized by a diverse ecosystem of microorganisms (oral microbiome) that maintains a balanced state through dynamic inter-microbial and host interactions. The oral microbiome is known to influence the hosts’ state of systemic and oral health or disease (Deo and Deshmukh, 2019). Although the oral microbiome has been shown to exhibit fluctuations depending on diet, circadian rhythm, and physiologic factors, a healthy microbiome demonstrates an ability to return to equilibrium (McBain et al., 2019).. When the oral microbiome is dysregulated, also known as dysbiosis, there is an increase in the abundance of periodontal pathogens such as *Porphyromonas gingivalis*, *Treponema denticola*, and *Tannerella forsythia*, infectious fungi (*Candida* spp.), or cariogenic bacteria (e.g. mutans streptococci and lactobacilli) (Nishikawara et al., 2007; Costalonga and Herzberg, 2014; Palmer, 2014; Montelongo-Jauregui and Lopez-Ribot, 2018). These dysbioses are associated with the development of periodontitis, dental caries, halitosis, and opportunistic mucosal infections (Costalonga and Herzberg, 2014; Singh et al., 2014; Lamont and Hajishengallis, 2015; Mogilnicka et al., 2020).

A key host mechanism in maintaining oral health and microbial stability is saliva, which plays a protective role by buffering the chemical and biological processes in the mouth, providing enzymatic activity, and serving as an inhibitor of pathogenic microorganisms (Amerongen and Veerman, 2002). In dry mouth (hyposalivation), the lack of salivary secretions and its antimicrobial properties can result in dysbiosis. For instance, Sjögren’s syndrome is a systemic autoimmune disease that is characterized by impaired salivation. The oral microbiota profile analysis of those with Sjögren’s syndrome has shown a significant difference from that of healthy patients, with increases in species indicated with dysbiosis (e.g. members of the red complex) and a decrease in *Porphyromonas pasteri*, which is associated with periodontal health (Rusthen et al., 2019). Higher levels of cariogenic bacteria and elevated caries prevalence has also been observed in these patients (Mathews et al., 2008; Tsigalou et al., 2018). Long-term mouth breathing may present similar challenges, where drying of the teeth and oral mucosa results in changes to the microbiome. Patients with this habit show greater counts of *Streptoccocus mutans* and lactobacilli (Mummolo et al., 2020), and higher incidence of oral malodor (halitosis) (Motta et al., 2011).

With the widespread use of masks for an extended period of time as a result of the COVID-19 pandemic, there has been anecdotal reports among healthcare providers and the public of xerostomia (subjective sensation of dry mouth), halitosis, and gingivitis after prolonged wear of mask, a consortia of symptoms colloquially termed “mask mouth” (Colgate Palmolive, 2022). However, the phenomenon has not been scientifically investigated. While a recent questionnaire-based study (Purushothaman et al., 2020) involving 250 healthcare workers showed that 35% and 22% of the participants reported experiencing symptoms of xerostomia and halitosis, respectively, the findings were not objectively validated, for example by measurement of salivary flow rate. To the best of our knowledge, there has been no attempts to study the effect of prolonged mask wearing on oral health.

Therefore, the aim of this study was to examine whether prolonged mask wearing indeed results in reduction of salivary flow rates (hyposalivation), alterations to microbial community composition, or shifts in gingival health status.

## Materials and methods

### Study design

The study was designed such as assessments were performed at three time points: T1, immediately before winter break where masks had been worn for extended hours daily for at least 2 months prior to the study; T2, immediately after the 2-3-week winter break assuming mask wear would be minimal and it would therefore serve as a “washout” period for microbiome reset; and T3, 2-3 weeks after resuming clinical practice with prolonged mask wearing again. The study was conducted between December 2020 and February 2021.

### Recruitment of study subjects

Twenty-five participants fulfilling the following criteria were recruited from among Junior and Senior dental students attending Temple University’s Kornberg School of Dentistry: involvement in full-day clinical affairs (at minimum 5 hours per day) while wearing masks (surgical or N95) over at least 2 months prior to the study; no evidence of candidiasis or opportunistic oral infections; no current oral abscess, ulcerations, or lesions caused by oral microbial agents; no history of antibiotic, antifungal, or steroid intake within 3 months prior to sampling; not on medications that cause dry mouth symptoms; not on triclosan or chlorhexidine mouthwash; no recent history of dental prophylaxis in the previous 30 days; systemically healthy as self-reported with no history of co-morbidities like diabetes, immunodeficiencies, or respiratory illnesses; and no diagnosis of periodontal disease.

This study was approved by the Institutional Review Board at Temple University (Protocol no. 27761), and conducted in accordance with the Helsinski declaration on medical research involving human subjects. Informed consent was obtained from all participants.

### Clinical examination and assessment of xerostomia

These were performed at each of the three time points (T1, T2 and T3). A questionnaire was used to collect information about mask use and associated subjective symptoms of dry mouth (xerostomia). The latter was evaluated as described by Fox et al. (1985), where each positive answer was scored as 1 and summed to give a final score out of 10, where a higher value indicates a greater sensation of dry mouth. Clinical examination included assessment of gingival health status using the gingival index (GI) (Loe and Silness, 1963) and plaque index (PI) (Silness and Loe, 1964), recorded at the six Ramfjords teeth (Ramfjord, 1959). Clinical examinations were performed by a single examiner.

### Collection of saliva and measurement of flow rate

Subjects were instructed not to eat or drink within 2 hours of sample collection. All samples were collected in the morning between 8 am to 12 pm. Unstimulated whole saliva (UWS) samples were collected at T1, T2, and T3 as detailed by Navazesh and Kumar (Navazesh et al., 2008). Briefly, subjects were asked to rinse their mouth with distilled water and rest for 5 minutes, after which they sat motionless and swallowed to void mouth of any saliva before drooling into a chilled plastic container for 5 minutes. Saliva was transferred to the laboratory on ice for measurement of UWS flow rate and subsequent microbial analysis. The former was calculated as follows:


$$Salivary\;flow\;rate=\;\frac{postweight\;measure-preweight\;measure\;\left(g\right)}{collection\;period\;\left(\text{min}\right)}$$

Where preweight measure is the weight of the empty container. The rate was then reported in ml/min with the assumption that 1 g of saliva corresponds to 1 mL (Johansson et al., 2012).

### Candida assay

Two-hundred μL aliquot of the freshly collected UWS was plated on CHROMagar™ Candida (BD, USA) and incubated for 48h at 30°C for enumeration (colony forming units per ml) of salivary *Candida* spp.

### DNA extraction

For DNA extraction, 500 µl of each UWS sample were mixed with 500 µL PBS containing 3.75 mM dithiothreitol and then centrifuged at 13000 rpm for 10 minutes to pellet the cells. Supernatant was discarded, and the pellets were each suspended in 162 µL phosphate-buffered saline and 18 µL of Metapolyzyme (Sigma, USA) and incubated at 35°C for 4 hours for digestion. DNA was subsequently extracted using PureLink™ Genomic DNA Mini Kit (Invitrogen, USA), according to manufacturer’s instructions. DNA concentration was determined by Qubit dsDNA HS kit (Invitrogen, USA).

### 16S rRNA gene sequencing and bioinformatic analysis

Library preparation and sequencing were done at the Integrated Microbiome Resource (IMR, Halifax, Canada). Briefly, the degenerate primers 27FYM (Frank et al., 2008) and 519R (Lane et al., 1985) were used to prepare indexed libraries of the V1-V3 region of the 16S rRNA gene that were subsequently sequenced on an Illumina Miseq platform using 2*300 bp chemistry.

Forward and reverse sequences were merged using PEAR (Zhang et al., 2014) and subsequent preprocessing of the merged reads including quality-filtration, alignment and chimera check was done using mothur software package version 1.39.5 (Schloss et al., 2009) as previously described (Al-Hebshi et al., 2017). The high-quality, non-chimeric sequences were classified to the species-level employing our BLASTN-based algorithm (Al-Hebshi et al., 2015; Al-Hebshi et al., 2017). A BIOM (Biological Observation Matrix) table was generated and used for downstream analysis with QIIME (Quantitative Insights Into Microbial Ecology) (Caporaso et al., 2010) including generation of taxonomy plots and calculation of species richness and diversity. Taxonomic read counts were centered log-ratio (CLR) transformed and used for principal component analysis (PCA) based on Aitchison’s distances using Phyloseq (McMurdie and Holmes, 2013) and Microbiome packages in R (Lahti and Shetty, 2017) and for differential abundance analysis using MaAsLin2 (Mallick et al., 2021). Selected significant results were plotted using ggplot2 package in R. Given the small sample size, no stratified analysis was done by mask type (surgical vs. N95).

## Results

### Demographics of the study population

Twenty-five junior and senior dental students participated in this study with a mean age of 26.36 ± 1.58 years: 12 were females, 13 were males. Of the participants, 16 identified as White, 8 as Asian, and 1 as African American.

### Prolonged mask wear did not affect oral health, xerostomia score or salivary rate

Consistent with our study design, there was a significant reduction in daily mask wear from 7.26 ± 1.56 hours at T1 to 1.13 ± 1.13 hours at T2, and then back to 6.93 ± 1.80 hours at T3 (P < 0.001). However, there were no statistical differences in xerostomia scores, UWS flow rate, or PI and GI across the three time points (**Table 1**). Overall, the scores were consistent with good oral health (low PI, GI and xerostomia scores, and normal salivary flow rate).

**Table 1:** Clinical characteristics by time point.

Variable	T1n=25	T2n=25	T3n=25	P value^*^
No. of mask hours	7.26 ± 1.56	1.13 ± 1.13	6.93 ± 1.80	0.000
Xerostomia score	3.40 ± 2.16	2.64 ± 2.06	3.30 ± 2.32	0.140
UWS flow rate (ml/min)	0.682 ± 0.406	0.662 ± 0.314	0.694 ± 0.374	0.738
Plaque index	0.46 ± 0.31	0.27 ± 0.16	0.27 ± 0.22	0.191
Gingival index	0.68 ± 0.25	0.67 ± 0.19	0.74 ± 0.22	0.083

^*^ Friedman test.

There were significant differences by gender (**Supplementary Table 1**). Namely, the females reported lower hours of mask usage but higher xerostomia score and had lower UWS flow rate. They also showed better oral hygiene (lower PI). For the purpose of comparison of variables by race (**Supplementary Table 2**), African Americans were excluded due to limited sample size (n=1). Hours of mask usage and xerostomia scores did not differ between the White and Asian groups, but the latter had lower salivary flow rate and higher GI scores.

### Salivary microbial profiles

**Figure 1:**
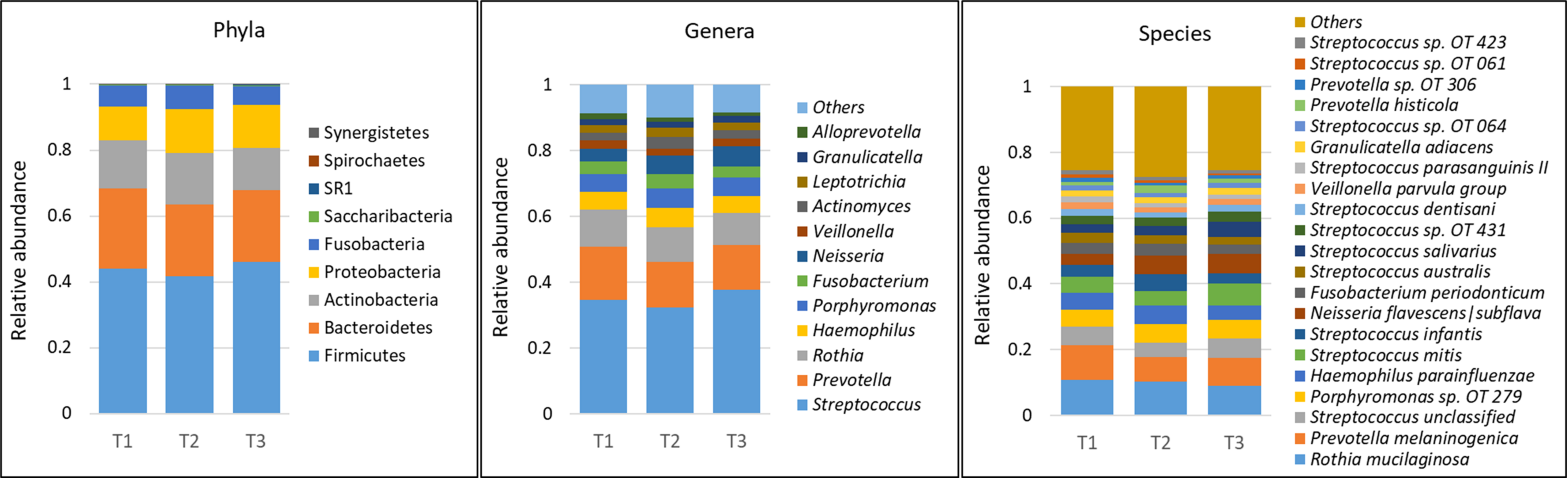
Microbial profiles by time point (mask wearing). DNA extracted from unstimulated saliva samples was sequenced for V1-V3 regions of the 16S rRNA gene using paired-end chemistry. Merged, quality-filtered were assigned species level taxonomies using a BLASTn-based algorithm. Stacked bars represent the average relative abundances of all the identified phyla across samples, and top genera and species.

A total of 232 species belonging to 63 genera and 9 phyla were detected in the samples – an average of 140 species and 47 genera per person. The average microbial profiles by time point, gender and race are shown in **Figure 1** and **Supplementary Figures 1A, B**, respectively. Regardless of grouping, the phyla Firmicutes, Bacteroidetes, Actinobacteria, Proteobacteria, and Fusobacteria, in this order of abundance, were most dominant and accounted for more than 99% of the average microbiome. Similarly, at the genus level, *Streptococcus, Prevotella, Rothia, Haemophilus, Porphyromonas, Fusobacterium, Neisseria*, and *Veillonella* were the most dominant and made up ~80% of the microbiome on average. At the species level, *Rothia mucilaginosa, Prevotella melaninogenica, Porphyromonas* sp. *oral taxon 279, Haemophilus parainfluenzae, Streptococcus mitis, Streptococcus infantis, Neisseria flavescens|subflava, Fusobacterium periodonticum, Streptococcus australis* and *Streptococcus salivarius* were the most abundant accounting for more than 50% of the microbiome on average.

The CHROMagar assay tested positive in 8 subjects (32%) in T1 and 5 subjects (20%) in T2 and T3. Only one subject tested positive in all three time points. The median CFU count was 15 CFUs/ml.

### Prolonged mask wear did not have a significant impact on the oral microbiome

**Figure 2:**
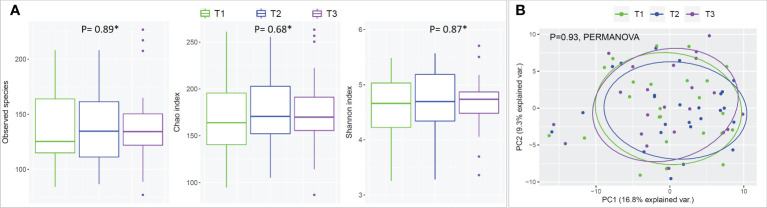
Species richness, alpha- and beta-diversity by time point (mask wearing). **(A)** Rarified taxonomic profiles were used to calculate observed species, Chao index and Shannon’s alpha diversity for each time point. * Statistical significance was assessed with Friedman's test. **(B)** Principle Component Analysis (PCA) plots were generated from centered log-ratio transformed taxonomic profiles.

**Figure 3:**
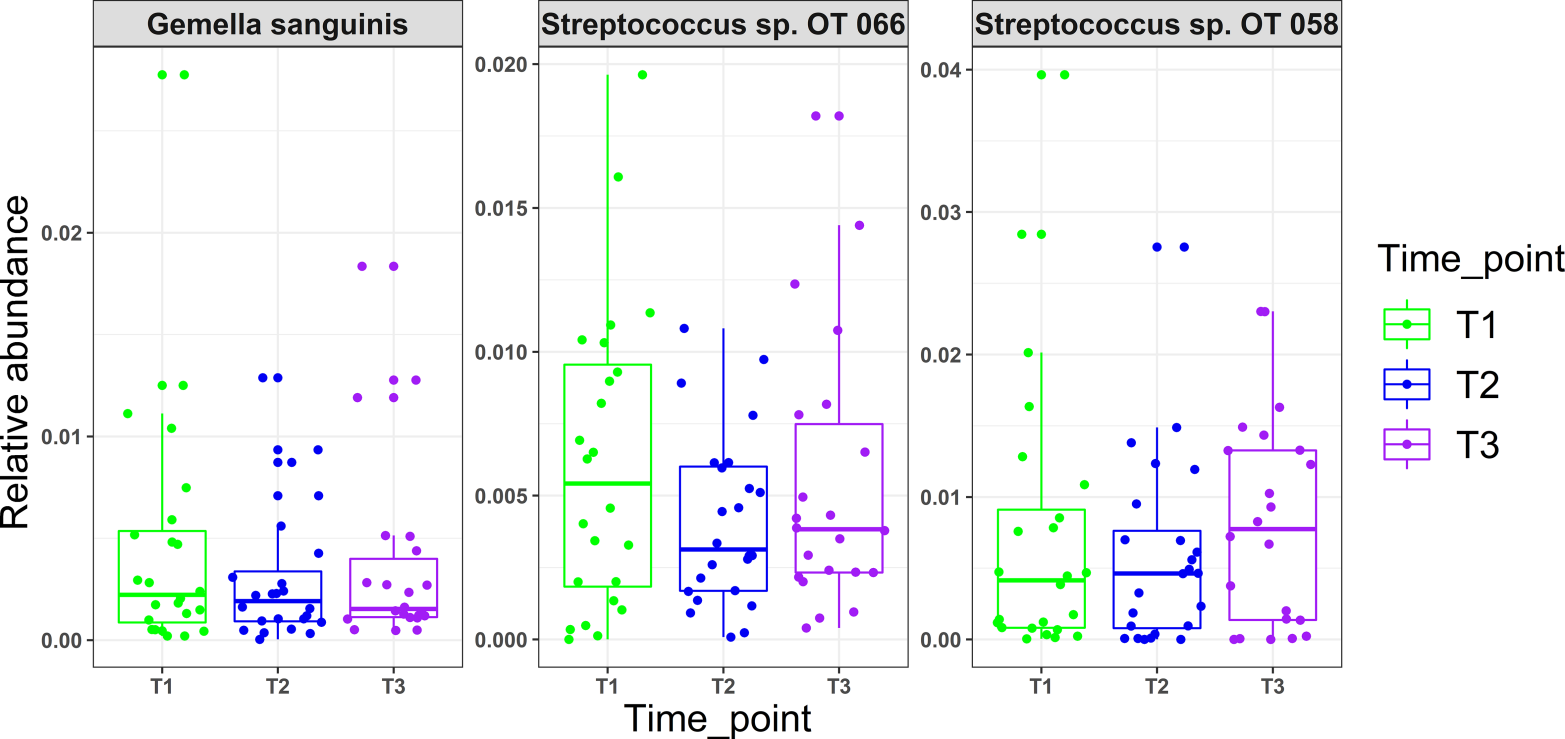
Microbial changes with mask wearing (time points). MaAsLin2 R package was used to identify significant associations with mask wearing adjusting for age, xerostomia score, salivary rates, GI and PI as fixed effects and time point as random effect. Normalization was performed using centered log-ratio transformation (CLR); nominal p values ≥ 0.01 were considered significant.

There were no statistical differences between samples across the three time points in species richness (observed and Chao index), alpha diversity (Shannon index) or beta diversity (PCA plot; PERMANOVA) as illustrated in **Figure 2**. Differential abundance analysis with MaAslin2 revealed no significant differences at the default discovery rate (FDR) of 0.1. However, using a nominal p-value of 0.01 as a cut-off identified some differences consistent with the study design, namely a decrease at T2 (followed by an increase in T3) in the abundances of *Gemella sanguinis* and *Streptococcus* oral taxa 58 and 66 **Figure 3**.

There were no statistically significant changes in the detection frequency of *Candida* spp. as a function of mask wearing by McNemar’s test (T1 vs. T2 and T2 vs. T3).

### Trends of microbial association with other study variables

There were trends of microbial associations with demographic and clinical characteristics independent of mask wearing. Males and females significantly differed in beta diversity but not in species richness or alpha diversity (**Supplementary Figure 2**). Differences by race were significant for beta diversity and species richness but not for alpha diversity. However, MaAsline analysis did not identify differentially abundant taxa when correcting for repeated measurements (random effect) at FDR of 0.1. Still, trends were observed at a nominal value of 0.01, including higher abundance of an unclassified *Fusobacterium species* and *Prevotella oris* in the females, *Actinomyces* oral taxon 175 and *Streptococcus anginosus* in the Asian subjects, and *Eikenella corrodens and Kingella dentitrificans* in the White subjects (**Supplementary Figure 3**).

Additional relevant associations were identified at a nominal p-value of 0.01. For example, an increase in age was associated with higher abundance of the periodontal pathogens *Porphyromonas gingivalis, Prevotella intermedia*, and *Fusobacterium nucleatum* but lower abundance of health-associated microflora (*Streptococcus salivarus, Leptotrichia o*ral taxon 417*, Alloprevotella* oral taxon 308), as shown in **Supplementary Figure 4**. An increase in GI was associated with a decrease in *Porphyromonas* sp. oral taxon 285 and *Firmicutes* oral taxon A55 but an increase in *Leptotrichia hongkongensis* while an increase in PI was associated with a decrease in *Butyrivibrio* oral taxon 455 and *Veillonella parvula* **Supplementary Figure 5**. With higher xerostomia score, a greater relative abundance of *Streptococcus* oral taxon 66 and *Lachnospiraceae* oral taxon 500 was observed, whereas *Granulicatella elegans* was reduced. An increase in salivary flow rate was associated with an increase in *Prevotella melaninogenica* and a decrease in *Streptococcus oralis* (**Supplementary Figure 6**).

## Discussion

This study was performed to address the emerging concerns that prolonged mask wearing may adversely affect oral health through what is called ‘mask mouth’. Namely, we assessed the effect of prolonged mask usage among dental students on subjective feeling of dry mouth (xerostomia), salivary flow rate, the oral microbiome and gingival health, which to the best of our knowledge is being done for the first time. The study design was challenging since ideally one would obtain baseline measurements from a group that had not worn masks and then follow it up after introducing mask usage. Naturally, however, that was not possible since healthcare workers routinely use masks as part of standard precautions that became even more reinforced during the COVID-19 pandemic. Therefore, as a compromise, we built the study design around the ~ 3-week winter break which we assumed would be a period of low mask wearing. Indeed, the student reported substantially lower daily mask usage (~ 1 hour) in T2 compared to T1 and T3 (~7 hours).

Typical daily production of saliva ranges from 0.5 to 1.5 liters, with whole unstimulated saliva flow rates approximating 0.3-0.4 ml/min (Iorgulescu, 2009). Disruptions in salivary gland function or hypsalivation (defined as when UWS is < 0.1 ml/min) can lead to dysbiosis which have been observed in individuals affected by Sjögren’s syndrome (Rusthen et al., 2019) or in mouth-breathers (Mummolo et al., 2020). Mask wearing has been reported to result in breathing difficulties (Li et al., 2005; Lee and Wang de, 2011) and introducing mouth-breathing (Kanzow et al., 2021), and thus it can be hypothesized that mask wearing can result in some level of mouth dryness. However, our findings did not show significant changes in UWS flow rate with increased hours of mask wear, and it was above the normal average of 0.3-0.4 ml/min at all time points. Similarly, there were not significant difference in xerostomia scores (as measured on a subjective perception scale adapted by Fox et al. (1985)) and the average score was low (3.11 out of 10), which is not consistent with the study by Purushothaman et al. (2020) in which 35% of the participant reported a feeling of dry mouth. The differences in findings may be attributed, at least in part, to the fact that the current study assessed xerostomia on a quantitative scale rather than as a dichotomus variable (yes/no response). It is important to note though that xerostomia is subjective complaint of dry mouth and does not necessarily equate to inadequate salivary production (Iorgulescu, 2009; Villa et al., 2015). Therefore, salivary flow rate is the most reliable measure of dry mouth.

Use of N95 or surgical facemasks have been shown to induce higher temperatures and humidity, creating a microclimate within masks (Li et al., 2005). With the fact that physical factors such as oxygen, moisture, and pH acting as driving forces in creating ecological niches for different microbial species (Human Microbiome Project, 2012), one would expect that mask wear would present similar challenges and thus induce compositional changes in the oral microbiome. However, there were no significant differences observed in oral microbiological profiles with prolonged mask use in this study. While using a relaxed significance cut-off (i.e., a nominal value of 0.01) revealed that *Gemella sanguinis*, *Streptococcus* sp. Oral taxon 066 and Oral taxon 058 tended to have lower abundances in T2 (low mask use) compared to T1 and T3 (prolonged mask use), the clinical relevance of these changes is questionable. A study by Roy et al. (2020), investigating the effect of prolonged medical mask use on nasal and oropharyngeal microbiota through culture-based methods similarly found no changes.

*Candida* spp., the most abundant members of the oral fungal community, also did not show any significant changes across the three time points. However, it is interesting to note that out of the eight subject’s positive for *Candida* spp. at T1, six became negative and the remaining two showed a reduction in fungal load, a trend worth of further investigation in a larger scale study, with more sensitive mycological analysis.

As with xerostomia, salivary flow rates, and microbiome profiles, we did not observe significant changes in gingival health because of prolonged mask wearing, for example lower PI and GI in T2 compared to T1 or T3. All subjects had minimal amounts of plaque with no to mild gingivitis (mean PI and GI values of < 1).

There were trends of microbiome differences by gender, race, salivary flow rate, xerostomia and age independent of mask wearing. While discussing these trends is beyond the scope of this paper, we would like to elaborate on the changes by age given their clinical relevance. Despite the narrow age range, older age was associated with an increase in the periodontal pathogens *Porphyromonas gingivalis*, *Prevotella intermedia*, and *Fusobacteria nucleatum* and a decrease in health-associated species, namely *Streptococcus salivarius, Leptotrichia* sp. Oral taxon 417, *Alloprevotella* sp. Oral taxon 308), which is interesting given risk of periodontitis increases with age. Consistently Rodenburg et al. (1990) found that *Prophyromonas gingivalis* increases with aging. Similarly, in analysis of periodontitis subjects of older adults by Feres et al. (2016), a significant elevation of *Prevotella intermedia* and a trend towards higher *Fusobacteria nucleatum* subspecies was noted. These finding suggests there is an increase in the levels of these pathogens before the onset of periodontitis.

In conclusion, the current study, using objective quantitative methods, found no evidence that prolonged mask wear among full-time dental students during the COVID-19 pandemic was associated with dry mouth or adverse oral health effect, the so called “mask mouth”. There were also no changes in the oral microbiome which substantiates the evidence that the oral microbiome in healthy individuals is resilient. However, the results may not be generalizable, since dental students are young and generally maintain good oral hygiene. It may well be that the effect of prolonged mask use is different in older subjects or in those with poor oral hygiene or existing gingivitis, which warrants evaluation in a future study.

## Data availability statement

The datasets presented in this study can be found in online repositories. The names of the repository/repositories and accession number(s) can be found below: https://www.ncbi.nlm.nih.gov/bioproject/879745.

## Ethics statement

The studies involving human participants were reviewed and approved by Temple University’s Institutional Review Board. The patients/participants provided their written informed consent to participate in this study.

## Author contributions

SA, JD, SBA, and JA conceived the study, contributed to the study design, collected the saliva samples, and contributed to the laboratory work and data entry. SA also drafted the manuscript. SS performed all clinical examinations. DB contributed the laboratory work, bioinformatic analysis, and writing of the manuscript. TC contributed to the bioinformatic analysis. SP contributed to the study design, interpretation of the data, overseeing the laboratory work, and critically revised the manuscript. NNA contributed to the study conception and design, analysis and interpretation of the data, critically revised the manuscript, and oversaw the execution of the project. All authors gave final approval and agreed to be accountable for all aspects of the work.

## Funding

This study was supported by an intramural fund from the Kornberg School of Dentistry, Temple University. Publication of this article was funded in part by the Temple University Libraries Open Access Publishing Fund and in part by Dr. Cary R. Klimen Oral Health Sciences Research Program Fund.

## Acknowledgments

We would like to thank Pavani Kondru for her help with collection of the samples.

## Conflict of interest

The authors declare that the research was conducted in the absence of any commercial or financial relationships that could be construed as a potential conflict of interest.

## Publisher’s note

All claims expressed in this article are solely those of the authors and do not necessarily represent those of their affiliated organizations, or those of the publisher, the editors and the reviewers. Any product that may be evaluated in this article, or claim that may be made by its manufacturer, is not guaranteed or endorsed by the publisher.
