# Supplementary material for: Prolonged mask wearing does not alter the oral microbiome, salivary flow rate or gingival health status – A pilot study
Source: Front Cell Infect Microbiol. 2022 Nov 10;12:1039811. doi: 10.3389/fcimb.2022.1039811 (PMC9684305; doi:10.3389/fcimb.2022.1039811)
Supplement: Supplementary file 1 [file DataSheet_1.docx]

**Supplementary tables and figures**

**Supplementary Table 1**

| **Variable** | **Males**  n=13 | **Females**  n=12 | **P value^*^** |
| --- | --- | --- | --- |
| No. of mask hours | 5.31±3.48 | 4.77±2.93 | 0.025 |
| Xerostomia score | 2.51±2.05 | 3.79±2.14 | 0.005 |
| UWS flow rate (ml/min) | 0.778±0.332 | 0.566±0.364 | 0.013 |
| Plaque index | 0.40±0.24 | 0.29±0.27 | 0.025 |
| Gingival index | 0.70±0.27 | 0.69±0.18 | 0.740 |

**^*^** Linear mixed-effects model analysis (time points as random effect)

**Supplementary Table 2**

| **Variable** | **Caucasian**  n=16 | **Asian**  n=8 | **P value^*^** |
| --- | --- | --- | --- |
| No. of mask hours | 5.15±3.41 | 5.01±2.89 | 0.341 |
| Xerostomia score | 3.04±2.35 | 3.13±1.94 | 0.681 |
| UWS flow rate (ml/min) | 0.756±0.388 | 0.532±0.274 | 0.042 |
| Plaque index | 0.30±0.27 | 0.39±0.23 | 0.091 |
| Gingival index | 0.63±0.25 | 0.78±0.14 | 0.020 |

**^*^** Linear mixed-effects model analysis (time points as random effect)


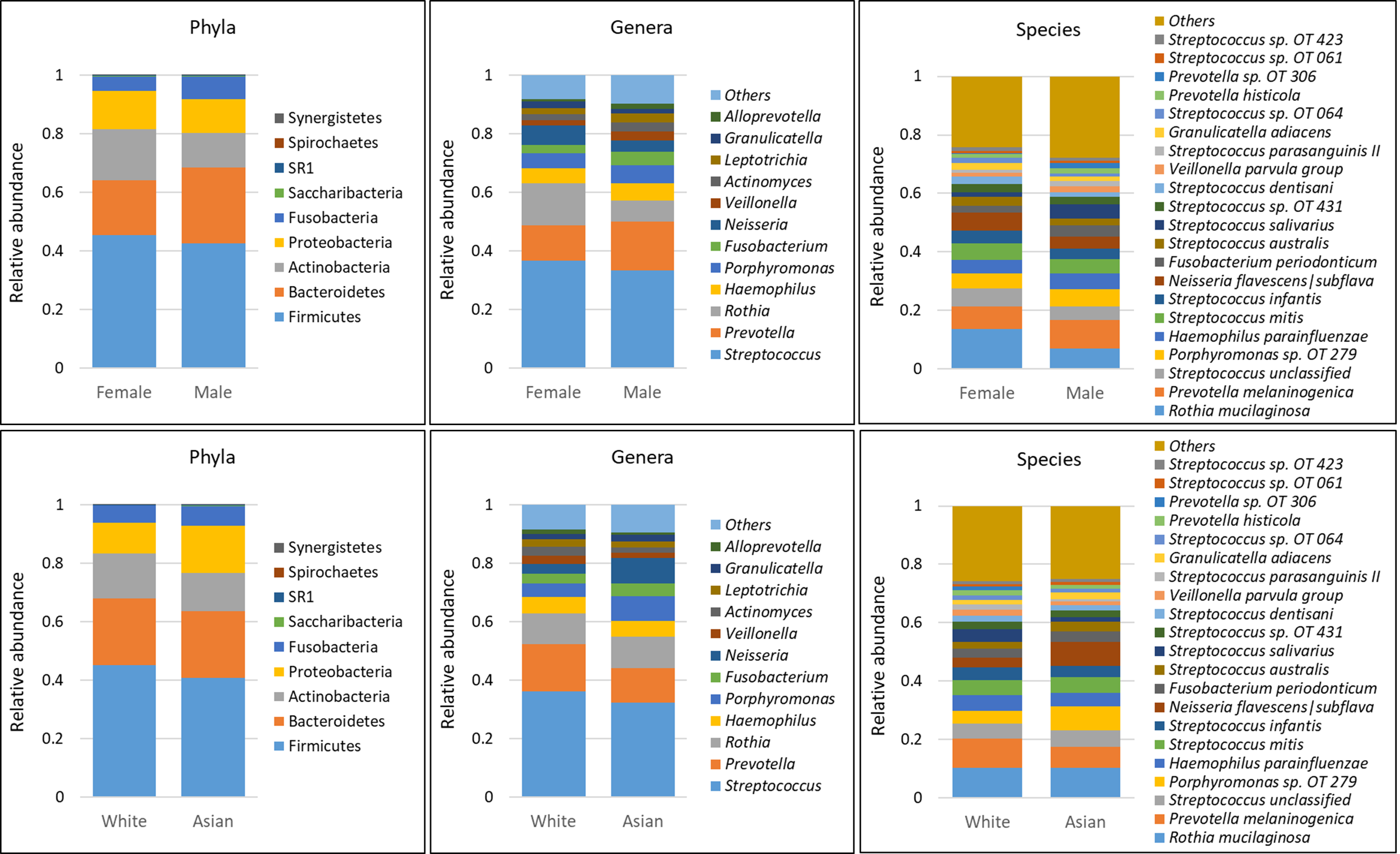


**Supplementary Figure 1. Microbial profiles by gender (top) and race (bottom).** DNA extracted from unstimulated saliva samples was sequenced for V1-V3 regions of the 16S rRNA gene using paired-end chemistry. Merged, quality-filtered were assigned species level taxonomies using a BLASTn-based algorithm. Stacked bars represent the average relative abundances of all the identified phyla across samples, and top genera and species.


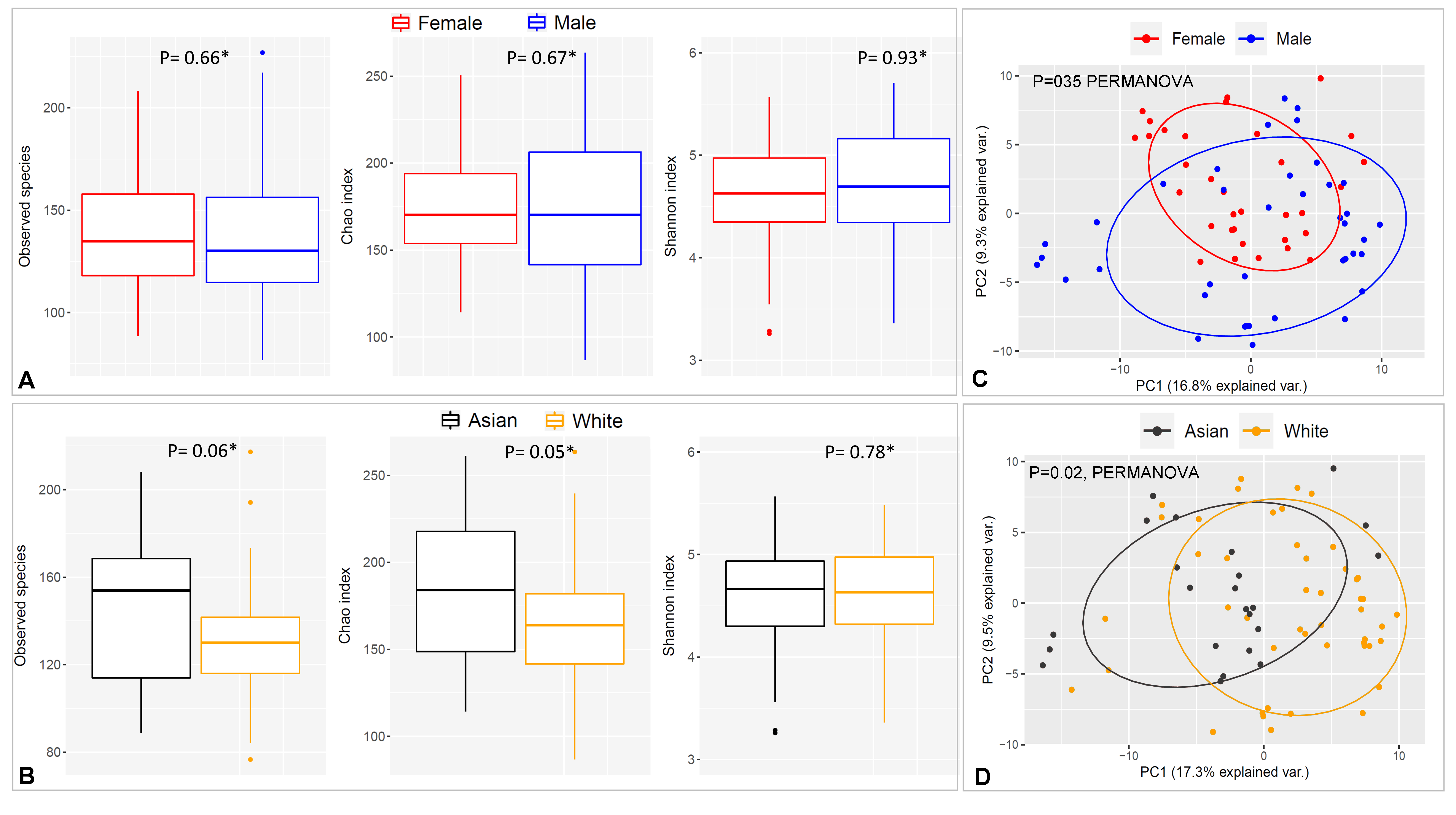


**Supplementary Figure 2. Species richness, alpha- and beta-diversity by gender and race**. Rarified taxonomic profiles were used to calculate observed species, Chao index and Shannon’s alpha diversity index for **A** males and females and **B** Asians and Whites. * Statistical significance was assessed with linear mixed effects model analysis. Principle Component Analysis (PCA) plots were generated from centered log-ratio transformed taxonomic profiles by **C** gender and **D** race.


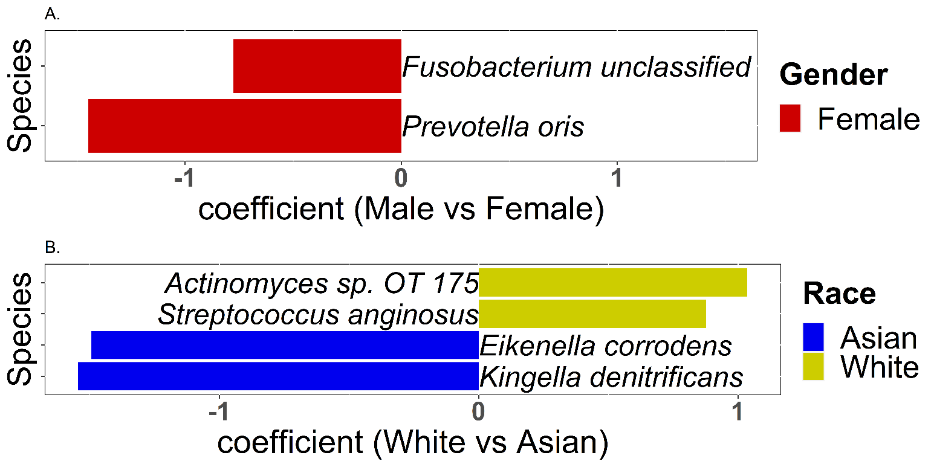


**Supplementary Figure 3. Significant microbial associations with gender and race**. MaAsLin2 R package was used to identify significant associations with gender and race adjusting for each other and for age, xerostomia score, salivary rates, GI and PI as fixed effects and time point as random effect. Normalization was performed using centered log-ratio transformation (CLR); nominal p values ≥ 0.01 were considered significant.


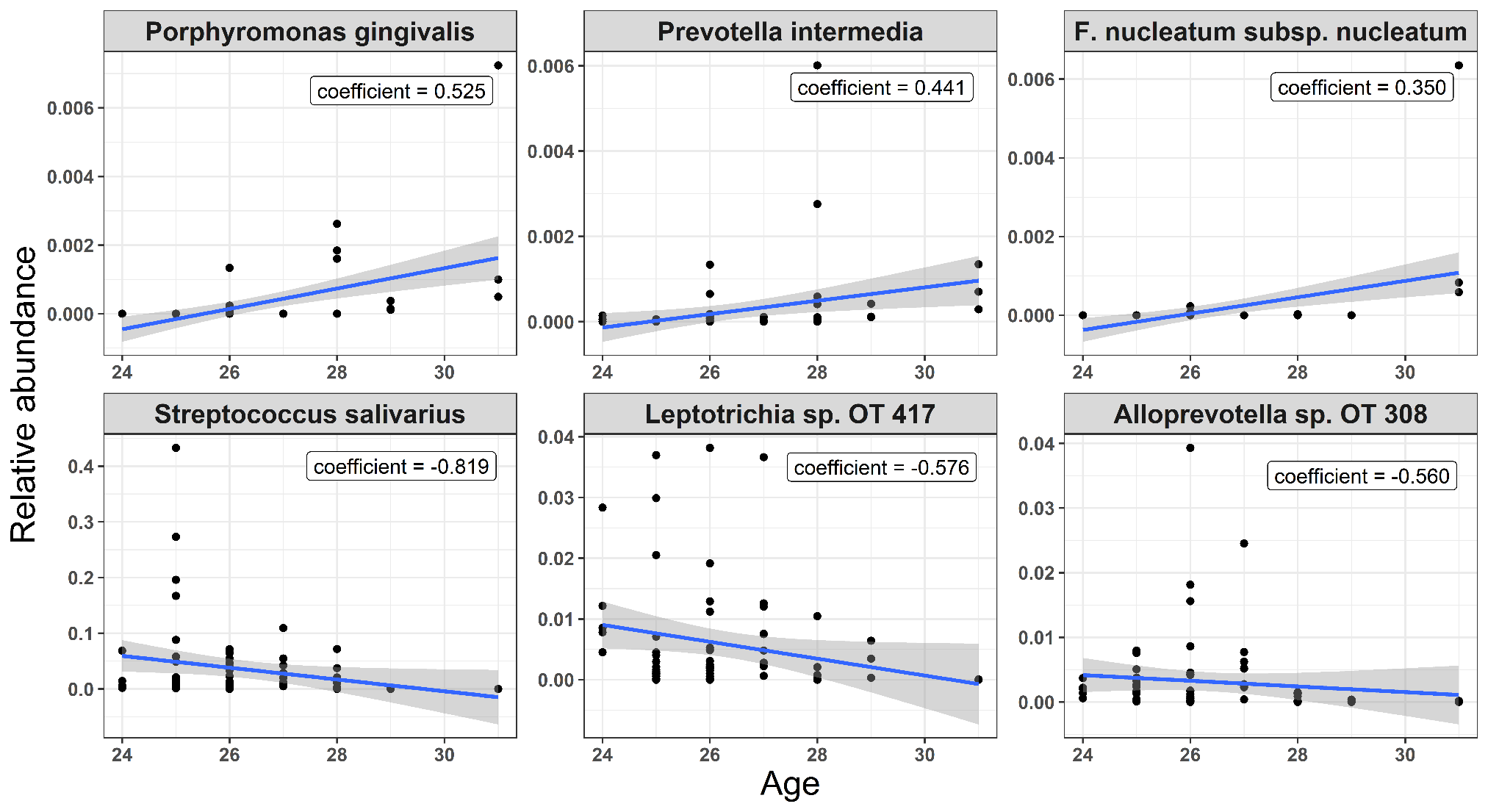


**Supplementary Figure 4. Significant microbial associations with age**. MaAsLin2 R package was used to identify significant associations with age adjusting for gender, race, xerostomia score, salivary rates, GI and PI as fixed effects and time point as a random effect. Normalization was performed using centered log-ratio transformation (CLR); nominal p values ≥ 0.01 were considered significant.


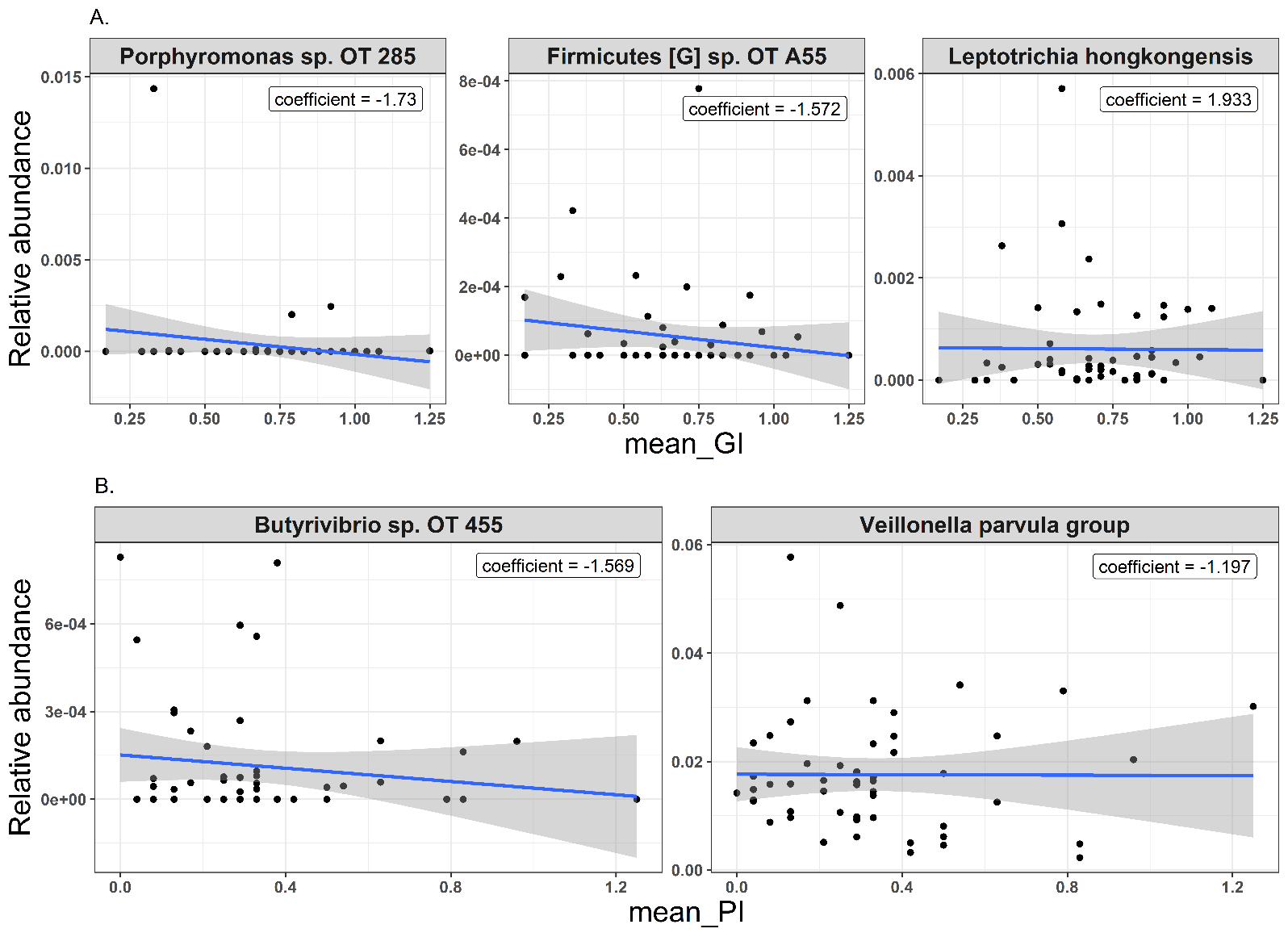


**Supplementary Figure 5. Significant associations with GI and PI**. MaAsLin2 R package was used to MaAsLin2 R package was used to identify significant associations with GI and PI adjusting for each other and for gender, race, age, xerostomia score, salivary rates as fixed effects and time point as a random effect. Normalization was performed using centered log-ratio transformation (CLR); nominal p values ≥ 0.01 were considered significant.


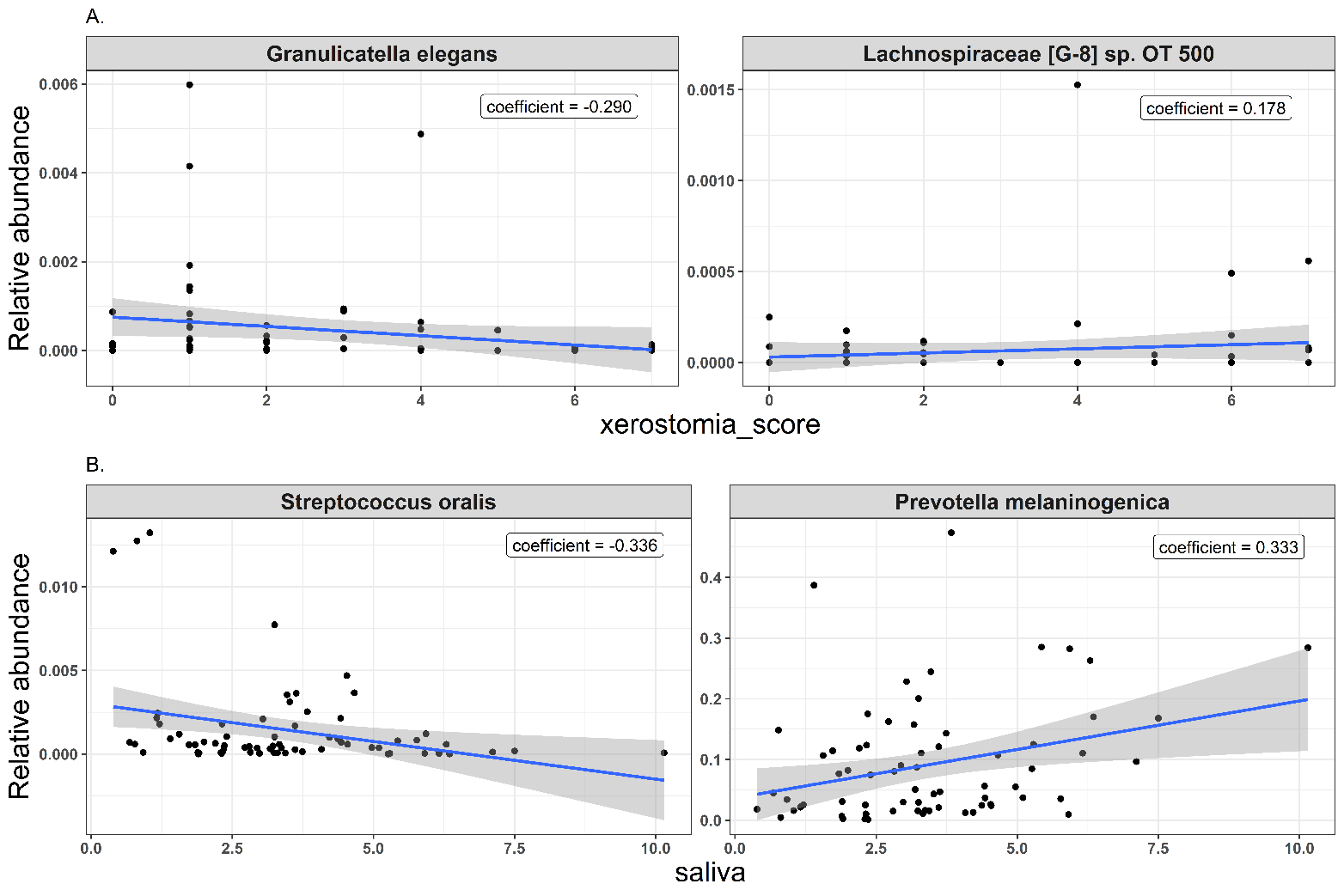


**Supplementary Figure 6. Significant associations changes with xerostomia score and salivary rates**. MaAsLin2 R package was used to identify significant associations with xerostomia score and salivary rates PI adjusting for each other and for gender, race, age, GI and PI as fixed effects and time point as a random effect. Normalization was performed using Centered Log Transformation (CLR) method p values ≥ 0.01 were considered significant.
